# A rare case of refractory hypoxaemia in a patient with large ostial right coronary artery thrombus and large atrial septal defect

**DOI:** 10.1093/ehjci/jead022

**Published:** 2023-02-15

**Authors:** Chrysovalantou Nikolaidou, Mina Fares, Saul G Myerson, Sam Dawkins

**Affiliations:** Division of Cardiovascular Medicine, Radcliffe Department of Medicine, University of Oxford, John Radcliffe Hospital, Headley Way, Headington, Oxford OX3 9DU, UK; Division of Cardiovascular Medicine, Radcliffe Department of Medicine, University of Oxford, John Radcliffe Hospital, Headley Way, Headington, Oxford OX3 9DU, UK; Division of Cardiovascular Medicine, Radcliffe Department of Medicine, University of Oxford, John Radcliffe Hospital, Headley Way, Headington, Oxford OX3 9DU, UK; Division of Cardiovascular Medicine, Radcliffe Department of Medicine, University of Oxford, John Radcliffe Hospital, Headley Way, Headington, Oxford OX3 9DU, UK

**Figure:**
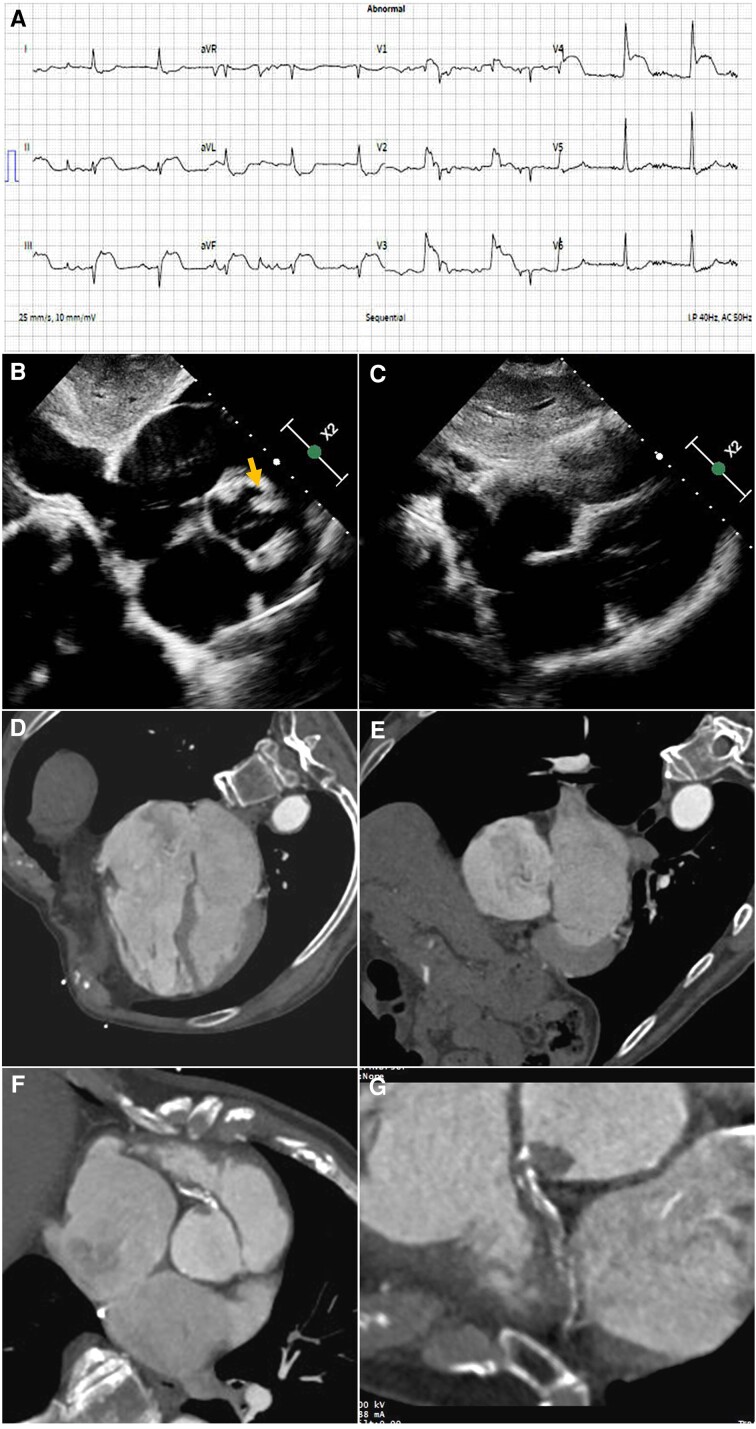


An 87-year-old male with background of hypertension and permanent atrial fibrillation presented after collapsing while watching cricket. He was peripherally cyanosed, with an oxygen saturation of 60% on 10 L/min oxygen. He did not report any chest pain. Blood pressure and heart rate were normal. Clinical examination was unremarkable, except for elevated jugular venous pressure. The electrocardiogram demonstrated sinus rhythm, with Q-waves and ST elevation in the inferior and septal leads (*Panel A*). Serum Troponin I was normal. The INR was in the low therapeutic range (2.0). A bedside echocardiogram revealed severely dilated right cardiac chambers with severe right ventricular (RV) systolic impairment and evidence of RV volume overload. There was a large atrial septal defect (ASD) with right-to-left flow and a hyperintense lesion at the right coronary artery (RCA) ostium (*Panels B* and *C*, Supplementary data online, Videos*S1* and *S*2). The computed tomography pulmonary angiogram showed an extensively calcified and proximally occluded RCA, with a large thrombus protruding from the ostium into the aorta (*Panels D–G*, Supplementary data online, Videos*S3*). Intervention for the thrombotic RCA occlusion was considered but this would have been immensely complex and the risk was felt to be too high. ASD closure was considered inappropriate given the severe RV systolic impairment and longstanding large shunt. The patient was therefore managed palliatively, treated with low molecular weight heparin, and died a few hours later. This is a rare case illustrating acute right-to-left cardiac shunt through a large ASD, after an RV infarction likely due to paradoxical embolus of the RCA.

Supplementary data are available at *European Heart Journal—Cardiovascular Imaging* online.

**Funding:** None declared.

**Data availability:** The data underlying this article are available in the article and in its online supplementary material.

## Supplementary Material

jead022_Supplementary_Data

